# Independent loss events of a functional *tetherin* gene in galliform birds

**DOI:** 10.1128/jvi.00803-23

**Published:** 2023-09-15

**Authors:** Veronika Krchlíková, Rishikesh Lotke, Isabell Haußmann, Markéta Reinišová, Dana Kučerová, Ľubomíra Pecnová, Lenka Ungrová, Jiří Hejnar, Daniel Sauter, Daniel Elleder

**Affiliations:** 1 Institute of Molecular Genetics of the Czech Academy of Sciences, Prague, Czech Republic; 2 Institute for Medical Virology and Epidemiology of Viral Diseases, University Hospital Tübingen, Tübingen, Germany; Icahn School of Medicine at Mount Sinai, New York, New York, USA

**Keywords:** retroviruses, restriction factors, gene loss, turkey, *tetherin*

## Abstract

The restriction factor tetherin (bone marrow stromal cell antigen 2) is an interferon-inducible protein preventing the release of newly formed viral particles from infected cells. Tetherin displays antiviral activity against a broad range of enveloped viruses, including retroviruses. While tetherin orthologs have been identified in several mammalian species, little is known about its expression and activity in non-mammalian vertebrates, including birds. We have previously described antiviral activity of chicken (*Gallus gallus*) tetherin against the prototypical avian retrovirus avian sarcoma and leukosis virus (ASLV). Here, we report the loss of functional tetherin orthologs in several galliform birds, including turkey (*Meleagris gallopavo*) and Mikado pheasant (*Syrmaticus mikado*). In both species, the *tetherin* coding sequence acquired inactivating mutations, including an in-frame stop codon and frameshifting deletions. Similar to the chicken tetherin ortholog, reconstituted turkey and Mikado pheasant tetherins restricted ASLV and HIV-1 indicating antiviral activity of the ancestor protein. Previous work revealed the presence of the *TMCC(aT*) gene in close proximity to tetherin, encoding a protein with tetherin-like structure in multiple vertebrates. Intriguingly, ectopic overexpression of chicken and turkey TMCC(aT) proteins in human cells decreased total HIV-1 yield. In contrast to tetherin, however, TMCC(aT) did not specifically restrict virion release, suggesting distinct antiviral mechanisms. In line with this, IFNα stimulation did not reduce the release of ASLV particles from infected turkey cells. Overall, our data describe the loss of functional *tetherin* genes in several galliform species and identify an antiviral activity of the related TMCC(aT) protein that is mechanistically different from that of tetherin.

**IMPORTANCE:**

Birds represent important hosts for numerous viruses, including zoonotic viruses and pathogens with the potential to cause major economic losses to the poultry industry. Viral replication and transmission can be inhibited or blocked by the action of antiviral restriction factors (RFs) encoded by the host. One well-characterized RF is tetherin, a protein that directly blocks the release of newly formed viral particles from infected cells. Here, we describe the evolutionary loss of a functional tetherin gene in two galliform birds, turkey (*Meleagris gallopavo*) and Mikado pheasant (*Syrmaticus mikado*). Moreover, we demonstrate that the structurally related protein TMCC(aT) exerts antiviral activity in several birds, albeit by a mechanism different from that of tetherin. The evolutionary scenario described here represents the first documented loss-of-tetherin cases in vertebrates.

## INTRODUCTION

Birds are natural hosts of a broad variety of pathogens, and it is widely accepted that they represent a potential natural reservoir of many known and unknown viruses with zoonotic potential. Notably, the global distribution of birds and their ability to fly over long distances enable them to spread viral infections during migration. They also frequently live in close proximity to human settlements, further increasing the risk of zoonotic transmission. For example, many strains of influenza viruses are hosted by waterfowl including ducks (1), while West Nile virus or various coronaviruses are carried by other avian species (2 – 5). Apart from zoonoses, galliform birds are also hosts of viruses causing drastic economic losses to the poultry industry, such as infectious bronchitis virus, Newcastle disease virus, or avian sarcoma and leukosis virus (ASLV) (6, 7). Notably, birds display specific adaptations of their immune system, which enable them to either coexist with viral pathogens or result in infections that are of low pathogenicity or asymptomatic in these species [reviewed in reference (8)].

Host cells have developed several sophisticated strategies for the detection and elimination of invading pathogens. These include the detection of viruses by pattern recognition receptors that subsequently trigger interferon (IFN) expression and the induction of interferon-stimulated genes (ISGs), which ultimately establish an antiviral state in the cell. Antiviral restriction factors (RFs) represent another important line of cellular defense. These cell-intrinsic proteins target specific steps of the viral replication cycle, thereby suppressing efficient viral replication and spread. While some RFs are constitutively expressed in several cell types, the expression of others is strongly upregulated by IFNs. To date, dozens of RFs have been identified in various vertebrates, but the antiviral activity of most of them has only been described in mammals. Despite the relevance of avian viruses for human and animal health, relatively little is known about the expression and antiviral activity of RFs in birds. Among the avian, RFs with known activity against retroviruses (e.g., ASLV) are death domain-associated protein 6 (DAXX), tripartite motif containing 62 and 25 (TRIM62 and TRIM25), and zinc finger antiviral protein (9 – 12). Apart from them, avian viperin, myxovirus resistance, interferon-induced protein with tetratricopeptide repeats 5 (IFIT5), and interferon-induced transmembrane proteins were previously shown to restrict viral replication (13 – 19).

One broadly active RF that has been characterized in detail in mammals, but not in birds, is tetherin, also known as bone marrow stromal cell antigen 2. Tetherin is an IFN-inducible type II transmembrane (TM) protein, whose topology comprises a short N-terminal cytoplasmic tail, followed by a TM domain, an extracellular coiled-coil (CC) domain, and a C-terminal glycosylphosphatidylinositol (GPI) anchor. Tetherin’s unique protein structure allows it to directly block the release of newly formed viral particles from infected cells by incorporating one of its membrane-associated domains into the envelope of the budding virion (20, 21). Due to its sequence-independent mode of action, tetherin displays antiviral activity against a broad range of enveloped viruses, including, e.g., retroviruses, herpesviruses, or coronaviruses (20 – 23). In response to the selection pressure exerted by tetherin, several viruses evolved antagonists, which are able to at least partially block tetherin’s activity, e.g., by inducing its removal from viral budding sites and/or degradation. Among the identified tetherin antagonists are the Vpu protein of HIV-1 (20, 21), Nef protein of SIVcpz (24, 25), and Env glycoprotein of HIV-2 (26).

Initially, tetherin was identified only in several mammalian species (25, 27, 28) leading to the assumption that tetherin is present just in eutherian mammals. The identification of tetherin orthologs in other vertebrate species was complicated by low primary sequence homology which reflects the importance of tetherin’s protein topology rather than primary amino acid sequence for its antiviral activity. As a consequence of this, new tetherin orthologs (including those from reptiles, birds, and fish) were identified based on their positions in the genome (between *CILP2* and *PLVAP* genes) and their conserved secondary protein structure rather than primary sequence homology (29, 30). Although tetherin was thought to be absent from several avian species (29), this was not confirmed in our previous work, where we identified *tetherin* orthologs in almost 70 avian species, including birds previously suggested to lack this gene (31). Additionally, we have identified chicken tetherin and demonstrated its antiviral activity against prototypic avian retrovirus ASLV (31).

Another gene located in close proximity to tetherin is *TMCC(aT*), also known as *CCDC194*. The name of this gene is based on the secondary structure of the respective protein, comprising a cytoplasmic tail followed by TM and CC domains and on its localization in the genome adjacent to *tetherin* (aT). The structural similarity of the three neighboring genes [*tetherin, TMCC(aT*), and *PLVAP*] suggests a common ancestor and continuous diversifying evolution (30). Blanco-Melo et al. showed that wild-type human and mouse TMCC(aT) proteins exert only limited antiviral activity but efficiently restrict HIV-1 release upon experimental addition of a C-terminal GPI anchor. Intriguingly, alligator and turtle TMCC(aT) variants inhibited the release of HIV-1 particles as efficiently as human tetherin, even without the addition of a GPI anchor signal at the C-terminus (30), indicating the presence of two genes with similar antiviral function in several vertebrate genomes.

In our computational screening of avian genomes for the presence of *tetherin* genes, we have previously found that the *tetherin* ortholog of turkey (*Meleagris gallopavo*) harbors a premature stop codon (31). Here, we characterize this loss-of-function mutation in detail. Furthermore, we analyze other members of the order Galliformes more broadly, as this order comprises both economically important species and relevant viral reservoir and carrier species. In addition to turkey, we report the loss of a functional *tetherin* gene in Mikado pheasant (*Syrmaticus mikado*). In both species, the coding sequence of tetherin acquired inactivating mutations including in-frame stop codons and frameshifting deletions. Furthermore, stimulation experiments revealed the absence of tetherin mRNA induction by interferon alpha (IFNɑ) in primary turkey cells. Similar to chicken tetherin, reconstituted turkey and Mikado pheasant tetherin efficiently restricted ASLV and HIV-1, indicating antiviral activity of the ancestor protein. Finally, we uncovered an antiviral activity of turkey and chicken TMCC(aT) orthologs. However, our experiments revealed that TMCC(aT) does not serve as a functional replacement of tetherin as it exerts a distinct mode of retroviral restriction.

## RESULTS

### Several galliform species harbor premature stop codons in their *tetherin* genes

**Fig 1:**
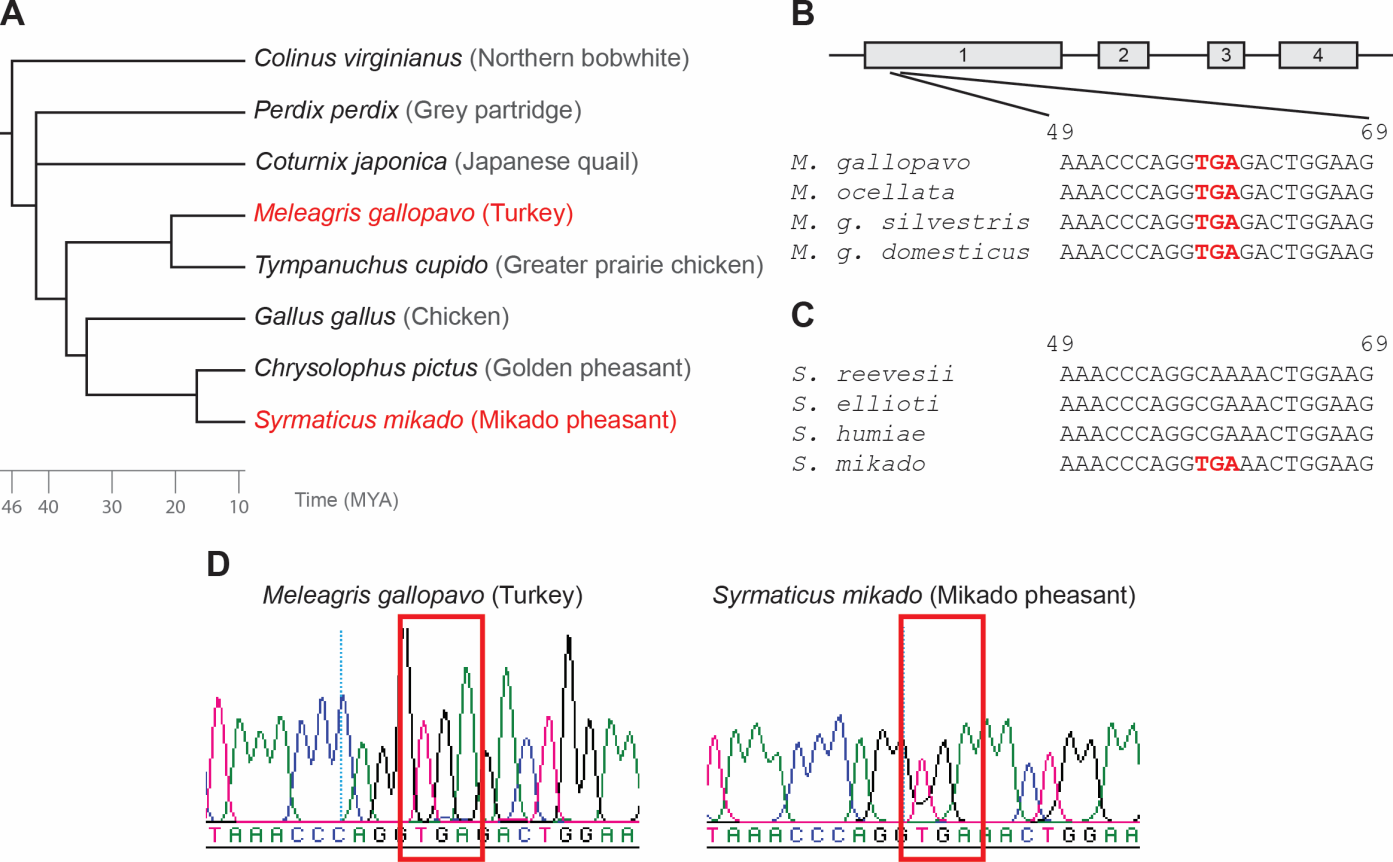
Identification of disrupted *tetherin* genes in Galliformes. (**A**) A time-calibrated phylogeny of several galliform species [TimeTree, (32)]; species harboring a disrupted *tetherin* gene are marked in red. (**B**) Sequence alignment of *tetherin* orthologs from *Meleagris* species with stop codons marked in red; in-scale exon-intron structure of tetherin is depicted above the alignments with exons marked by gray boxes. (**C**) Sequence alignment of *tetherin* orthologs from *Syrmaticus* species with stop codons marked in red. (**D**) Chromatograms of turkey and Mikado pheasant tetherin sequences amplified from genomic DNA with the stop codons marked by red rectangles; MYA, million years ago.

First, we identified tetherin sequences from various galliform species (Fig. 1A) based on sequence homology with previously described avian tetherin sequences (29 – 31) from publicly available sequencing data deposited at NCBI. We identified an in-frame stop codon in two galliform birds, *Meleagris gallopavo domesticus* (domestic turkey) and *Syrmaticus mikado* (Mikado pheasant). Despite their evolutionary distance, both species harbor the stop codon at the same position in the first coding exon of the *tetherin* gene (Fig. 1B and C). Next, we examined the tetherin coding sequences of closely related species of the *Meleagris* and *Syrmaticus* genera. To this end, we PCR-amplified the *tetherin* genes from genomic DNA of the two extant *Meleagris* species, *M. gallopavo* and *M. ocellata*, including two *M. gallopavo* subspecies, *M. g. silvestris* and *M. g. domesticus*. These sequence analyses revealed the presence of the same stop codon in all *Meleagris* species analyzed (Fig. 1B). Moreover, *tetherin* of all turkey species contains a 7-nucleotide deletion further downstream in the coding sequence of the first exon (not shown). The presence of the same mutations in all turkey species suggests that tetherin was inactivated in the common ancestor of these birds, approximately 5–21 million years ago (MYA). The amplification and sequencing of four distinct *Syrmaticus* species, i.e., *S. mikado, S. humiae, S. ellioti,* and *S. reevesii*, revealed that only *S. mikado* harbors the premature stop codon, while other *Syrmaticus* species encode an intact exon 1 (Fig. 1C). This suggests that Mikado pheasant tetherin disruption occurred after the speciation of *Syrmaticus* genus, within the last 3 million years. Notably, sequence chromatograms (Fig. 1D) and the analysis of illumina genome sequencing reads (data not shown) excluded heterozygosity of the inactivating mutations and showed no evidence for functional alleles in any substantial proportion of the sequences. Taken together, we identified three galliform species with disrupted *tetherin* coding sequences.

### Reconstituted galliform *tetherins* display antiviral activity

**Fig 2:**
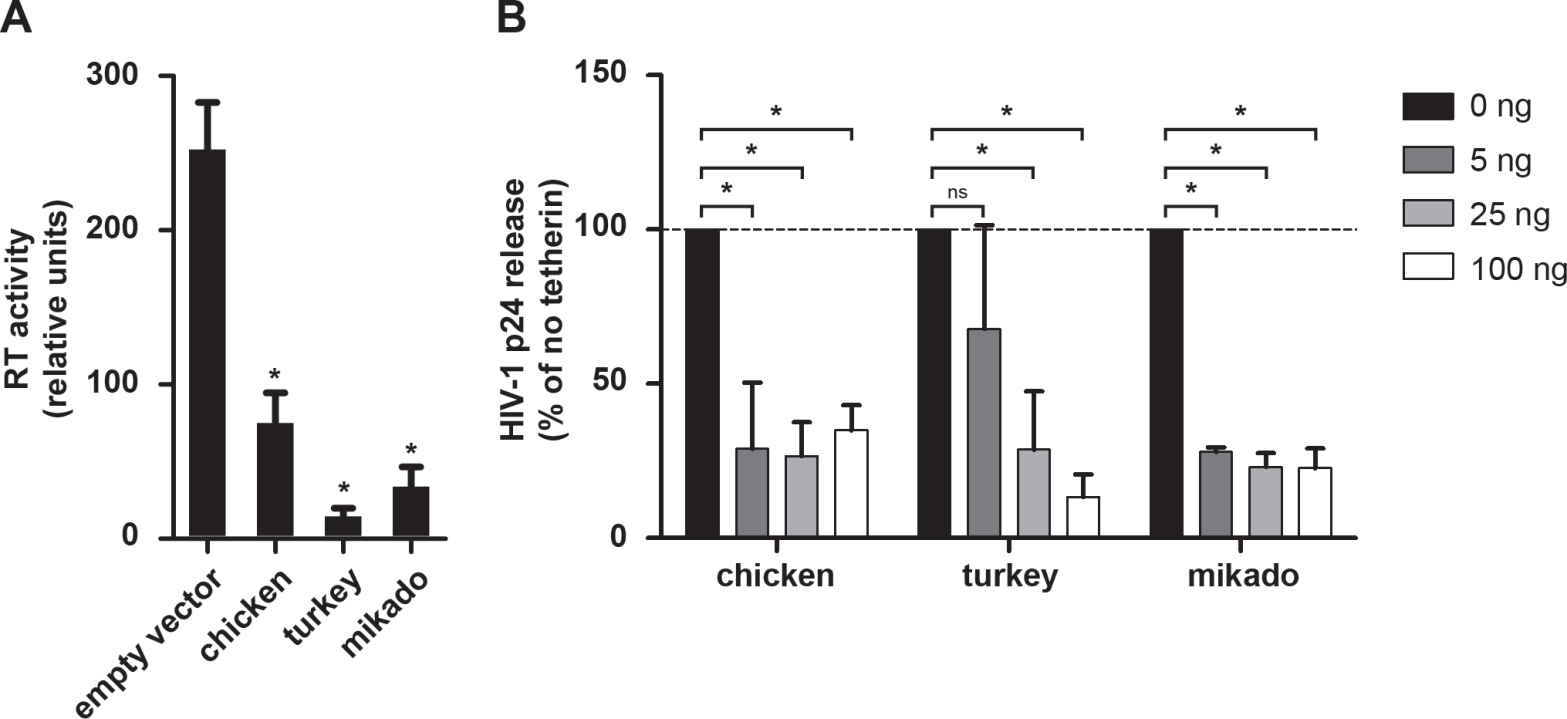
Antiviral activity of reconstituted galliform tetherins. (**A**) Chicken DF1 cells were transfected with the indicated tetherin expression constructs together with an ASLV-A vector. Two days post transfection, RT activity in the culture media was measured by PERT assay. (**B**) Human HEK293T cells were co-transfected with increasing amounts of the indicated tetherin expression constructs and the HIV-1 NL4-3 clone harboring a *vpu* gene with a premature stop codon. Two days post transfection, the amount of viral capsid protein (p24) in cells and supernatants was measured by ELISA. Relative virion release was calculated by normalizing the amount of viral capsid protein in the supernatants to that in the cells. Means and standard deviations of three independent biological replicates are shown; *P < 0.05.

To determine if the ancestral tetherins of turkeys and Mikado pheasants potentially displayed antiviral activity, we reconstituted turkey and Mikado pheasant tetherin according to the chicken *tetherin* sequence. This included the reversion of the premature stop codon and reversion of the frameshift mutations in the turkey ortholog. The 7-nucleotide deletion in the first exon of turkey tetherin was filled in by the corresponding nucleotides (CGGTCCA) found in chicken tetherin. The reconstituted turkey and Mikado pheasant tetherin sequences were deposited to GenBank under accession numbers OQ835635 and OQ849771, respectively. Chicken DF1 cells were then co-transfected with the respective expression plasmids for reconstituted turkey or Mikado pheasant tetherin and the RCASBP(A) vector expressing replication-competent ASLV subgroup A. Two days post transfection, restriction of retroviral particle release was measured by product-enhanced reverse transcriptase (PERT) assay. Similar to the previously characterized tetherin ortholog from chicken (31), reconstituted tetherins of both, turkey and Mikado pheasant, efficiently reduced RT activity in the cell culture supernatant (Fig. 2A). Notably, however, a direct comparison of the antiviral activities of these tetherin orthologs is difficult since expression levels could not be determined due to the lack of suitable antibodies. To exclude a potential antiviral effect masked by an unknown avian tetherin antagonist encoded by ASLV, we tested if the galliform tetherins are able to restrict a heterologous retrovirus, i.e., HIV-1. To this end, HEK293T cells were co-transfected with a pIRES-based tetherin expression plasmid and a *vpu*-deficient HIV proviral vector. The extent of virion release was measured 2 days post transfection by ELISA quantification of the viral capsid protein (p24) concentrations in the cells and supernatants. Both reconstituted turkey and mikado pheasant tetherin restricted the release of HIV-1 particles as efficiently as chicken tetherin, which was used as a control (Fig. 2B). Taken together, the reconstituted galliform tetherins displayed antiviral activity against ASLV and HIV.

### Expression of endogenous turkey *tetherin* is not induced by IFNɑ

**Fig 3:**
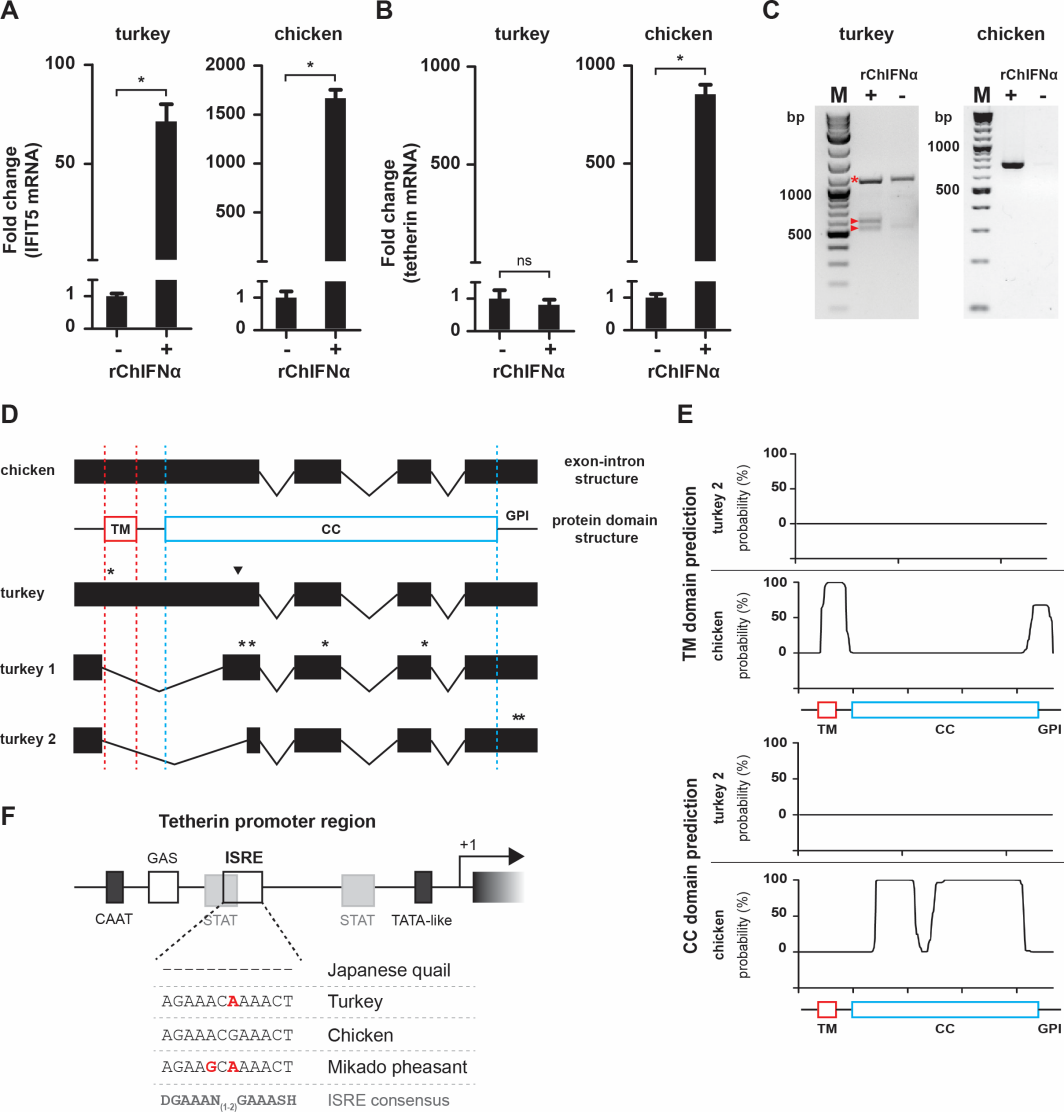
Turkey tetherin expression and structure. Turkey and chicken embryonal fibroblasts were seeded and treated with 2,000 U/mL of rChIFNɑ for 2 days. Expression of IFIT5 (**A**) and tetherin (**B**) was determined by qPCR. Means and standard deviations of three independent biological replicates are shown; *P < 0.01; (**C**) Analysis of splice isoforms of turkey tetherin. Turkey and chicken tetherin coding sequence was amplified from cDNA stimulated or not with 2,000 U/mL rChIFNɑ. The bands representing two alternative splice variants of turkey tetherin (turkey 1 and turkey 2) are marked by arrowheads, and amplified genomic DNA is marked by an asterisk. In the case of chicken tetherin, no genomic DNA was amplified, potentially because of different gDNA/cDNA ratios or different primer sets. rChIFNɑ, recombinant chicken interferon alpha; bp, base pairs; M, molecular marker. (**D**) Analysis of chicken and turkey tetherin splice variants. Premature stop codons are marked by asterisks and a frameshift deletion is indicated by black arrow head. (**E**) Prediction of the secondary protein structure of chicken tetherin and turkey tetherin splice variant 2. (**F**) Analysis of tetherin promoter regions from galliform birds. Basic promoter regulatory regions (CAAT and TATA box), IFN responsive elements (ISRE and GAS) and STAT binding sites are shown. Transcription start is depicted by an arrow. Substitutions supposedly disrupting the ISRE consensus are marked in red; TM, transmembrane domain; CC, coiled-coil domain; GPI, glycosylphosphatidylinositol anchor.

To further characterize the loss of the *tetherin* open reading frame in turkeys, we explored if turkey *tetherin* mRNA levels are upregulated by IFNɑ, as they are in chicken, even though the mRNA does probably not encode a functional full-length protein with tetherin-like activity anymore. For this purpose, we took advantage of primary turkey and chicken embryonal fibroblasts and stimulated them with recombinant chicken IFNɑ (rChIFNɑ). Importantly, expression of *IFIT5*, a well-characterized ISG (17), was induced in both chicken and turkey cells (Fig. 3A). Although turkey IFIT5 was induced less efficiently than chicken IFIT5 (most likely due to species-dependent effects of chicken IFNɑ), these findings confirm the effectiveness of IFNɑ stimulation in both species. In stark contrast, tetherin was only upregulated in chicken but not in turkey cells (Fig. 3B). Since turkey tetherin was expressed at low levels in the presence and absence of IFN, we amplified the respective transcripts from cDNA of turkey embryonal fibroblasts. Intriguingly, we identified two distinct bands after PCR amplification (Fig. 3C). Sequence analyses revealed that these bands represent two alternative splice variants of turkey tetherin (named turkey 1 and turkey 2; GenBank accession numbers OQ835633 and OQ835634, respectively), in which a newly identified splice donor in the center of exon 1 (next to the premature stop codon) is common for both variants, while the splice acceptor is different for each variant (Fig. 3D). As a result of this splicing pattern, both turkey tetherin isoforms lose their N-terminal transmembrane domain and—as predicted by *in silico* analyses—their coiled-coil domain (Fig. 3E) and GPI modification site. Moreover, the alternative splicing of both variants leads to a frameshift mutation and, thus, the introduction of premature stop codons (Fig. 3D). To further determine the range of inactivating mutations, we investigated the promoter regions of galliform tetherins witfh a focus on the Interferon-Stimulated Response Element (ISRE) which is crucial for IFNɑ stimulation (33). We identified ISRE elements in the galliform tetherin promoters based on the already described ISRE in the promoter of chicken IFIT5 (17). Surprisingly, our analysis revealed substitutions in the ISRE of several galliform birds, including turkey and Mikado pheasant, which are not consistent with the ISRE consensus sequence (Fig. 3F and data not shown). Thus, turkey tetherin not only acquired several inactivating mutations and splice sites that resulted in the loss of a typical tetherin ortholog but also accumulated potentially deleterious mutations in its ISRE sequences.

### Turkey TMCC(aT) proteins modestly inhibit HIV production

**Fig 4:**
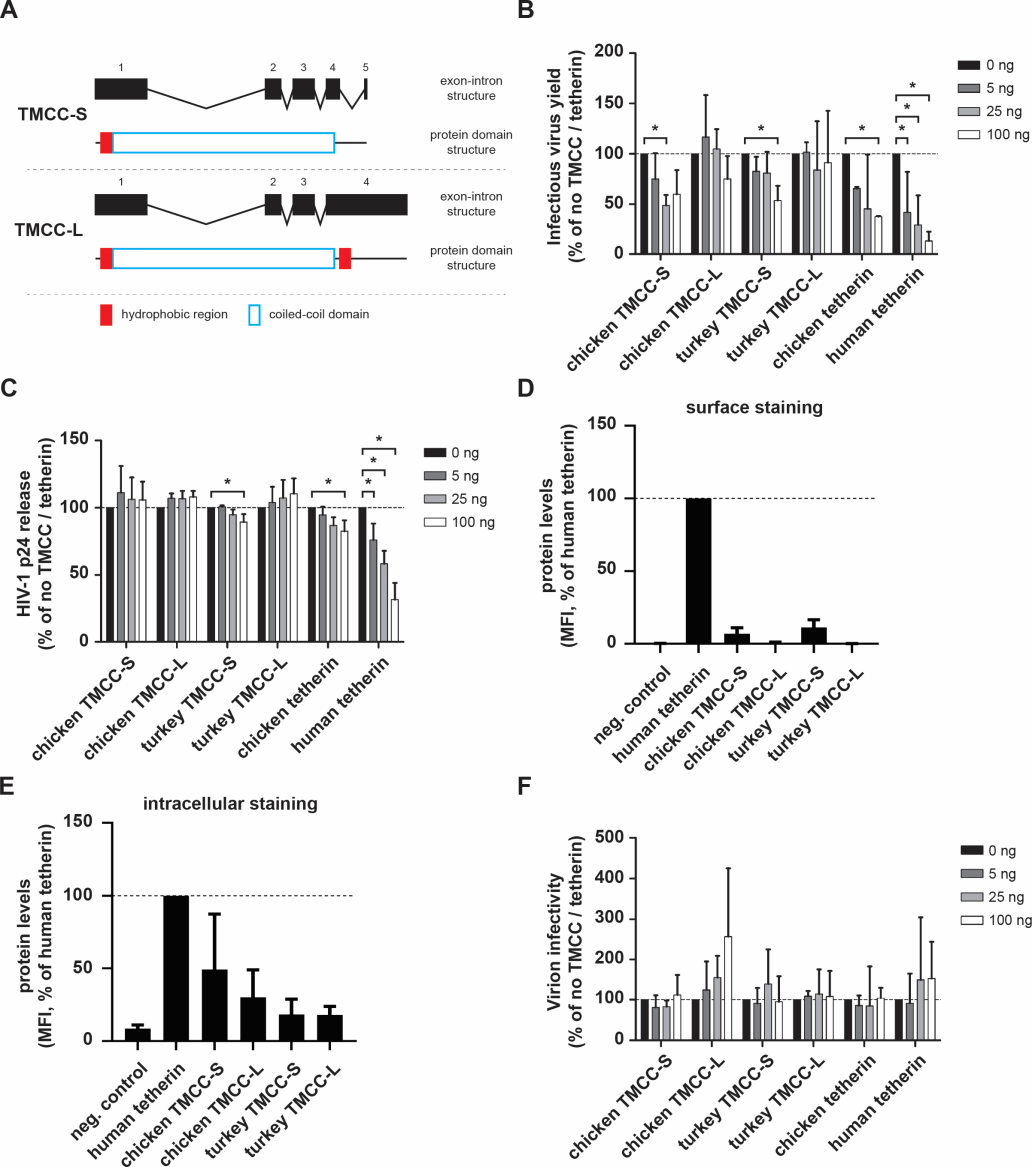
Antiviral activity of avian TMCC(aT) proteins. (**A**) Analysis of TMCC(aT) splice variants. The predicted exon-intron structure of TMCC(aT) isoforms is depicted in-scale with black boxes representing exons. The predicted protein structure is illustrated below each isoform. (**B and C**) HEK293T cells were co-transfected with expression plasmids for the indicated TMCC(aT) variants and a *vpu*-defective HIV-1 vector. Infectious virus yield (**B**) and p24 release (**C**) were measured 2 days post transfection. (**D and E**) Analysis of protein expression of TMCC(aT) variants. HEK293T cells were transfected with individual TMCC(aT) variants or human tetherin. One day post transfection, protein levels were determined by flow cytometry using an anti-Flag antibody for detection of surface (**D**) or intracellular (**E**) protein; (**F**) Relative virion infectivity was measured 2 days post transfection of HEK293T cells. The cells were co-transfected with expression plasmids for the indicated TMCC(aT) and a *vpu*-defective HIV-1 vector. Means and standard deviations of three independent biological replicates are shown; *P < 0.05.

The observation that turtle and alligator orthologs of the tetherin-like protein TMCC(aT) restrict HIV-1 (30) raised the possibility that TMCC(aT) proteins may act synergistically with tetherin or compensate for its loss in some species such as turkeys. To address this, we identified *TMCC(aT*) orthologs of chicken and turkey (using publicly available RNAseq data sets as described in the Materials and Methods) and cloned two possible TMCC(aT) isoforms for each ortholog. One isoform is truncated due to a splicing event from exon four to exon five (hereafter referred to as short isoform: TMCC-S), while the second isoform (hereafter referred to as long isoform: TMCC-L) continues through exon four and encodes for another hydrophobic region. Thus, this long isoform is predicted to adopt a topology that is similar to that of tetherin, where both ends harbor a membrane-associated domain (Fig. 4A). However, in contrast to tetherin (Fig. 3B and C), we did not detect *TMCC(aT*) expression in chicken or turkey primary fibroblasts (data not shown). To assess the antiviral activity of the individual TMCC(aT) isoforms, we co-transfected HEK293T cells with the respective TMCC(aT) expression plasmids and a proviral HIV-1 NL4-3 vector harboring a disrupted *vpu* gene. Two days post transfection, infectious virus yield and particle release were measured by the infection of indicator cells and p24 capsid ELISA, respectively. Both, chicken and turkey TMCC-S decreased the amount of infectious viral particles to about 50%, while the long isoforms did not display any significant antiviral activity in our experimental setup (Fig. 4B). Intriguingly, neither TMCC(aT) variant specifically affected the release of the newly formed virions from the cell in contrast to chicken or human tetherin (Fig. 4C). Nevertheless, we observed both chicken and turkey TMCC-S at the cell membrane even though at a lower amount compared to human tetherin (Fig. 4D). In contrast, TMCC-L proteins were only detected intracellularly (Fig. 4E). Furthermore, it was previously suggested that tetherin might affect the infectivity of newly formed virion particles (34, 35 ). However, HIV particle infectivity was not reduced by either TMCC(aT) isoform in our experimental setup (Fig. 4F). This is in agreement with other studies demonstrating tetherin’s inability to alter virus particle infectivity (36, 37). Taken together, our results suggest that TMCC(aT) employs a distinct mode of antiviral activity in comparison to tetherin.

### Turkey cells lack the late block of ASLV production

**Fig 5:**
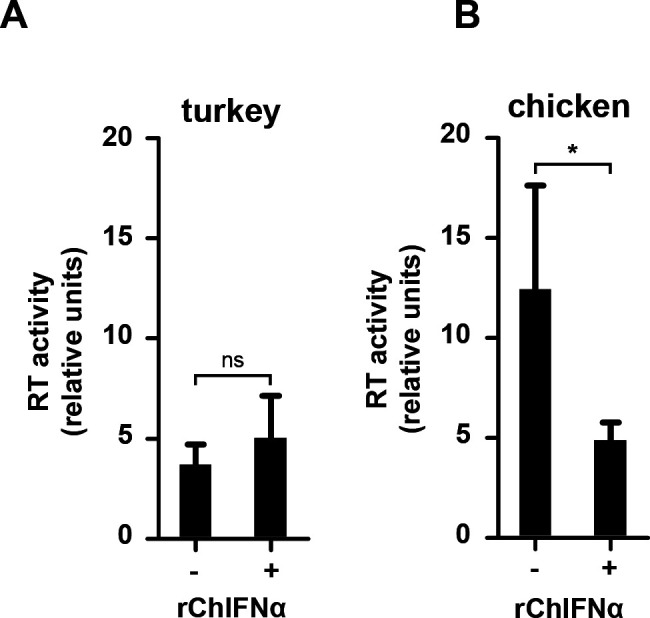
Block of ASLV release in chronically infected cells. Turkey (**A**) and chicken (**B**) embryonal fibroblasts were chronically infected with ASLV-A. Two weeks post infection, the cells were stimulated with 2,000 U/mL rChIFNɑ. Two days later, RT activity in the media was determined by PERT assay. Means and standard deviations of three independent biological replicates are shown; *P < 0.05.

To investigate whether any other protein may compensate for the lack of a normal tetherin ortholog in turkeys, we investigated the presence of a potential IFNɑ-induced late block of retroviral replication in turkey and chicken embryonal fibroblasts. In order to quantify the production and release of retroviral particles, we infected both cell types with the ASLV vector RCASBP(A)GFP and let the virus spread. Then, these chronically infected cells were stimulated with rChIFNɑ or not, and 2 days later, ASLV particle production and release were quantified by PERT assay. As expected, we detected reduced RT activity in chicken cells upon rChIFNɑ stimulation but did not observe any late block in chronically infected turkey cells (Fig. 5). This finding is in agreement with the expression of a functional tetherin ortholog in chicken (31) and the absence of an antivirally active tetherin in turkeys.

## DISCUSSION

In this study, we present the loss of the antiviral restriction factor tetherin in turkey and Mikado pheasant, which occurred independently at least twice during the evolution of galliform birds. The *tetherin* coding sequence of both species contains multiple inactivating mutations including a stop codon and frameshifting deletions (Fig. 1 and 3). Intriguingly, the premature stop codon is present in a homozygous state at the same position in both species despite their evolutionary distance. This suggests that the substitution did not originate in the common ancestor but occurred independently at identical sites in both species. Our results further suggest that tetherin in the common ancestor of galliform birds displayed antiviral activity against enveloped viruses since reconstituted turkey and Mikado pheasant tetherins efficiently restricted the release of retroviral particles (Fig. 2). This further supports the theory of Heusinger et al. that tetherin evolved more than 450 MYA, but functional tetherin orthologs might have been lost independently in several vertebrate species during evolution (29). The loss of this restriction factor in turkeys and Mikado pheasant could have been caused by the loss of evolutionary pressure, the exploitation of tetherin by some ancient virus, or its functional replacement by another gene.

As mentioned above, the loss of tetherin has been proposed before in several vertebrates including birds, amphibians, and fish (29, 31). Until now, vertebrate tetherin orthologs have not been identified in frogs (*Xenopus* sp.) or platypus (*Ornithorhynchus* sp.) (30). However, the lack of identification of a functional tetherin ortholog in these species does not imply that the gene is not present in their genomes. Instead, the lack of identification may be due to the low coding sequence homology, its absence in the conserved genomic locus, or the incompleteness of the genome assemblies. Intriguingly, disruption of the tetherin coding sequence has not been documented before. Thus, we are the first to present the independent loss of tetherin during vertebrate evolution, which occurred at least twice during the speciation of galliform birds. Interestingly, the loss of other restriction factors has also been described. For example, Mx1 and Mx2 are present in most mammals (38) but have been lost in Odontocetes (e.g., orcas and dolphins) (39). Another example is the loss of the entire family of PYHIN proteins in bats, which includes both nucleic acid sensors and restriction factors (40).

Notably, a predicted turkey tetherin coding sequence had already been published before (30 ). However, the published sequence lacks the initial start codon, as it begins just downstream of the stop codon described in this work (Fig. 1 and 3). Furthermore, its antiviral activity had not been experimentally tested. Despite the disruption of the prototypical tetherin ORF in turkeys, two tetherin splice variants are still detectable in primary turkey cells (Fig. 3). These splice variants harbor truncated ORFs that may encode for 30 and 122 amino acid proteins, respectively. Notably, however, potentially encoded proteins are unlikely to restrict virion release as they lack the N-terminal TM domain and most likely also the typical coiled-coil sequence. Moreover, tetherin mRNA levels are low in primary turkey cells and not increased upon IFNɑ stimulation (Fig. 3B). In contrast, human and chicken tetherin are both strongly upregulated upon IFNɑ treatment (20, 31). The human tetherin promoter harbors an ISRE that is essential for stimulation by IFNα (41). Intriguingly, our investigation of the galliform tetherin promoter revealed substitutions in the ISRE of turkey tetherin that disrupts the ISRE consensus sequence (Fig. 3F). It is tempting to speculate that these mutations resulted in the loss of IFN responsiveness. Intriguingly, we observed similar ISRE mutations in multiple other galliform birds, including Mikado pheasant, and several passeriform birds (data not shown), suggesting the loss of IFN inducibility of tetherin in multiple avian species. However, the reasons for the repeated occurrence of these substitutions in avian tetherin promoters are still unknown. It may be possible that tetherin is still induced by other cytokines, also in the absence of an ISRE. Human tetherin expression, for example, can also be induced by IL-27 (42). It remains to be determined whether disruption of the tetherin ORF in turkey and Mikado pheasants preceded the loss of its IFN responsiveness or *vice versa*.

The loss of a gene during evolution could be accompanied by its functional replacement by another similar gene. Strikingly, such an evolutionary event has been documented before, as the loss of HERC5 in mice was functionally compensated by HERC6 (43). Inspired by the findings described in Blanco-Melo et al. (30), we tested if the *tetherin* disruption in turkey led to its functional replacement by its neighbor, *TMCC(aT*). It was previously reported that TMCC(aT) proteins from several reptiles restricted HIV-1 in tetherin-like manner, while the mammalian orthologs had no effect on HIV-1 replication (30). Thus, the authors suggested that TMCC(aT) proteins of some species might be functional paralogs of tetherin. In line with this, the short isoforms of avian TMCC(aT) proteins reduced the total infectious yield of HIV-1 in human cells. In contrast to tetherin, however, TMCC-S expression did not affect virion release indicating a different mode of restriction (Fig. 4). Thus, it will be of interest to investigate if the avian TMCC-S orthologs inhibit LTR-driven viral gene expression, reduce translation of viral mRNA, decrease virion production, or employ a distinct mechanism to limit virus propagation. Even though both proteins (tetherin and TMCC-S) encode a transmembrane and a coiled-coil domain, TMCC-S lacks a GPI anchor at its C terminus which could explain the distinct antiviral activity. Similarly, different antiviral modes of action have been proposed for GPI-deficient tetherin paralogs from sheep (35) and bats (44). More specifically, ovine oBST2B reduces retroviral particle infectivity by decreasing virion incorporation of Env, while isoform C of *Pteropus alecto* tetherin restricts the release of Marburg VLPs but not HIV. In line with the results described above, the lack of an IFN-induced late block of ASLV replication in turkey cells (Fig. 5) strongly suggests that turkey tetherin is not functionally replaced by another protein, at least in the cell type tested. This is in agreement with our previous results, where we observed almost no late block of retroviral replication in the chronically infected chicken cells with *tetherin* knockout (31). This resemblance suggests that tetherin is one of the major antiviral factors in birds acting at the late stage of the antiviral replication cycle. These data further support our theory that the disrupted turkey tetherin is probably not fully replaced by TMCC(aT) although the antiviral activity of TMCC(aT) needs to be studied in more detail in the future.

Also beyond innate immunity, the expression pattern and physiological role of TMCC (aT) remain largely unclear. Compared with tetherin, expression of TMCC(aT) has not been investigated in detail and has been detected only in mouse embryos (30) and in the human upper respiratory tract during non-coronaviral acute respiratory illness (45). Notably, expression of TMCC(aT) in humans has also been associated with the expression of the non-coding RNA BISPR (46). Consistent with limited expression *in vivo*, we did not observe TMCC(aT) expression in galliform fibroblasts that were or were not stimulated with IFNɑ (data not shown). However, we cannot exclude the possibility that TMCC(aT) is expressed in other cell types, during distinct developmental stages or after stimulation with certain cytokines.

It is well known that wild birds represent natural reservoirs of multiple pathogens, including zoonotic viruses such as influenza virus or Newcastle disease virus (8). Interactions of wild birds with domestic animals, including poultry, may lead to the transmission of viruses between the two populations and potentially huge economic losses. Notably, tetherin not only poses a barrier to virus transmission within but also between different species (27, 47). Thus, it will be of interest to determine if the loss of tetherin in wild and domestic birds, such as turkeys and Mikado pheasants, led to higher transmission rates and/or increased susceptibility of these species to various enveloped viruses. Recently, epidemiological monitoring of lymphoproliferative disease virus (LPDV), a retrovirus infecting various fowl species, revealed a rapid spread of this virus in North American wild turkey populations (48 – 50). It remains to be determined if the loss of tetherin in turkeys contributed to the extensive dissemination of LPDV in this species.

Taken together, the losses of functional tetherin orthologs in turkey and Mikado pheasant are the first documented loss-of-tetherin cases in vertebrates. In addition, we demonstrate antiviral potential of the protein encoded by its neighboring gene TMCC(aT), which exhibits antiviral activity against retroviruses. However, the exact step of the retroviral replication cycle that it intercepts requires further research. It also remains open to what consequences the loss of tetherin in some birds has on their susceptibility to various enveloped viruses.

## MATERIALS AND METHODS

### Cells and viruses

The chicken fibroblast cell line DF1, embryonal fibroblasts of brown leghorn chicken and turkey were grown in a mixture of two parts of DMEM and one part of F-12 medium (Sigma-Aldrich) supplemented with 4% fetal calf serum, 4% calf serum, and 1% chicken serum, 100 µg/mL streptomycin and 100 U/mL penicillin under 5% CO_2_ atmosphere at 37°C. RCASBP(A)GFP and RCASBP(A)dsRED are replication-competent retroviral vectors based on ASLV subgroup A (51).

HEK293T and TZM-bl cells were cultured as described previously (52) in Dulbecco’s modified Eagle medium (DMEM) supplemented with FCS (10%), l-glutamine (2 mM), streptomycin (100 µg/mL), and penicillin (100 U/mL). TZM-GFP cells were cultured in DMEM supplemented with FCS (10%), l-glutamine (4 mM), 1 mM sodium pyruvate, streptomycin (100 µg/mL), and penicillin (100 U/mL). HEK293T and TZM-bl cells were provided and authenticated by the ATCC, whereas TZM-GFP cells were obtained through the NIH HIV Reagent Program, NIAID, NIH: TZM-GFP Human Cell Line (JC.53 Derived), HRP-20041, contributed by David G. Russell and David W. Gludish. All cells were cultured at 37°C, 90% humidity, and 5% CO_2_.

### Identification of tetherin and TMCC(aT) orthologs

To reconstruct avian tetherin and TMCC(aT) sequences, we used publicly available SRA reads from NGS sequencing deposited at the NCBI. The reads originating from avian tetherin and TMCC(aT) orthologs were identified by BLASTn searches using previously described avian tetherin or TMCC(aT) sequences as baits (53). The collected reads from each species were then assembled using DNASTAR Lasergene SeqMan Pro software. Transmembrane helices were predicted from primary protein sequences using TMHMM v2.0 (54) at https://services.healthtech.dtu.dk/services/TMHMM-2.0/. Coiled-coil regions were predicted with the algorithm of Lupas et al. (55) at https://npsa-prabi.ibcp.fr/NPSA/npsa_lupas.html. Predicted GPI modification sites were identified using server https://mendel.imp.ac.at/gpi/gpi_server.html (56) or https://busca.biocomp.unibo.it/predgpi/ (57).

### Cloning of turkey and pheasant *tetherin* and TMCC(aT) orthologs

Turkey and Mikado pheasant *tetherin* coding sequences with repaired stop codon and frameshift mutations and *TMCC(aT*) short and long isoforms were synthesized (Integrated DNA technologies). The synthesized oligonucleotides were subcloned into the pGEM vector and verified by Sanger sequencing. The plasmids were consequently digested by BamHI and XhoI (for *tetherin*) or BamHI and EcoRI [for *TMCC(aT*)] restriction enzymes (NEB) and subcloned into the pIRES2-EGFP vector (BD Biosciences Clontech). While tetherin orthologs were left untagged, we inserted an internal Flag tag in the TMCC(aT) proteins. The sequences of turkey and Mikado pheasant tetherins with repaired stop codons were deposited at GenBank under accession numbers OQ835635 and OQ849771, respectively. The sequences of codon-optimized Flag-tagged TMCC variants were also deposited at GenBank and are available under the following accession numbers: chicken TMCC-S (OQ849772) and TMCC-L (OQ849773); turkey TMCC-S (OQ849774) and TMCC-L (OQ849775).

### RNA isolation and qPCR

Total RNA was isolated from cultured cells using RNAzol RT (Molecular Research Center). Two micrograms of RNA was then reversely transcribed using Protoscript II reverse transcriptase (NEB) and the SMART RACE protocol (ClonTech). The qPCR reaction included cDNA sample with MESA GREEN qPCR MasterMix Plus (Eurogentec) and primers targeting GAPDH (chicken—5′CATCGTGCACCACCAACTG and 5′CGCTGGGATGATGTTCTGG, turkey—5′CCTCGTGCACCACCAACTG and 5′CCAGAACATCATCCCAGCA), IFIT5 (chicken—5′TGCTTCACCAGCTAGGACTCTGC and 5′TGGCTTTTGCTCTGTCACCACTTTG, turkey—5′AGAGGTTTTGGAGAGAGCCCT and 5′TTTGCTCAGTCACCGCTTTGA), tetherin (chicken—5′CAACAGGGCTCTCCAGGAGG and 5′GGCATCTCTGTGCTCCCACT, turkey—5′CAACAGGGTGCTCCAGGAG and 5′GCATCGCTGTGCTCCCACT), and TMCC(aT) (chicken—5′GCGAGAGGGCTGAGCTGTCC and 5′CTGCTCCGCCGTCTCCTCCA, turkey—5′GCGTGACAGGGCTGAGCTGT and 5′CTGCTCCGCCATCTCCTCCAG). The samples were run on a CFX96TM real-time instrument (BioRad) with a three-step protocol (1 cycle of 8 min at 95°C followed by 40 cycles of 15 s at 95°C, 25 s at 60°C, and 35 s at 72°C. Cycles of quantification (Cq) values were generated by the CFX Manager software, and the specificity of the PCR products was confirmed by melting curves analysis.

### PCR analysis of avian *tetherin*

Samples for PCR amplification were isolated from cultured embryo fibroblasts. Embryonated eggs for fibroblast derivation were obtained from Phasanerie Möller, Erfurt, Germany (*Syrmaticus mikado, S. humei, S. ellioti, Meleagris gallopavo silvestris*) and University of Veterinary and Pharmaceutical Science, Brno, Czech Republic (*Meleagris gallopavo domesticus*). DNA from ocellated turkey (*Meleagris ocellata*) was isolated from the feather pulpa. The feathers were obtained from Aves Farm, Košice, Czech Republic. Chicken and turkey embryonic fibroblast cells have been described before (58). The full-length *tetherin* coding sequence of turkeys was amplified from cDNA samples of primary turkey cells using 5′GCTCACAGCTTTTCTGGGTGA and 5′GGCAAACCATCTCCTCCTGGT primers and TDV polymerase [mix of Taq and Deep Vent polymerase 200:1 (NEB)] and the following PCR conditions: 35 cycles (25 cycles for the chicken samples) of 95°C for 20 s, 61°C for 30 s, and 65°C for 1 min followed by 10 min final polymerization. The fragments were separated on 2% agarose gel, and the individual bands were then excised using QiaEX Gel extraction kit (Qiagen) and sent for Sanger sequencing (SeqMe).

The whole genomic sequence of *tetherin* from various *Meleagris* sp. and *Syrmaticus* sp. was PCR-amplified from DNA samples of individual species and subspecies using turkey (5′GCTCACAGCTTTTCTGGGTGA and 5′GGCAAACCATCTCCTCCTGGT) and pheasant (5′CTGCTGCCCTTGAACCAATCAC and 5′CTGAACGTGCCTTGTCCCATCT) primers and TDV polymerase. The amplified fragments were then submitted for Sanger sequencing.

### PERT assay

We used the PERT assay for the quantification of virus particle release as described before (31). Briefly, virus-containing media were lysed in lysis solution [M-MLV 5 × buffer (Promega), Triton X-100, and RNAsin (Promega)] at room temperature for 15–30 min. Then, the two mastermixes were prepared: mix A containing MS2 RNA (Roche) and reverse primer (5′GCCTTAGCAGTGCCCTGTCT) was incubated at 65°C for 5 min and cooled to room temperature; mix B containing dNTPs and M-MLV buffer. The mixes were then pooled and aliquoted, and the sample lysates were added. The reaction was incubated at 37°C for 60 min and inactivated at 70°C for 10 min. The newly generated MS2 cDNA was then quantified by real-time PCR, each reaction mixture containing cDNA sample with qPCR MasterMix Plus, MS2 primers (forward 5′AACATGCTCGAGGGCCTTA and reverse primer mentioned above), and MS2 probe [6-carboxyfluorescein (FAM)-TGGGATGCTCCTACATG-6-carboxytetramethylrhodamine (TAMRA)]. The samples were run on a CFX96 instrument with a three-step protocol: 1 cycle of 10 min at 95°C and then 40 cycles of 15 s at 95°C, 20 s at 60°C, and 20 s at 72°C. Cq values were generated by the CFX Manager software.

### TZM-bl and TZM-GFP reporter assays

HEK293T cells were transiently transfected using the calcium-phosphate method as described previously (52). Virus stocks were generated by transfecting cells with 5 µg of *vpu*-deficient HIV-1 proviral plasmid per well of a six-well plate. To test the antiviral effect of Galliform tetherins or Galliform TM-CC(aT)s, pIRES-based expression constructs were co-transfected with the proviral plasmid. Since a dosage range of the Tetherin or TM-CC(aT) plasmid DNA was used to test for a dose-dependent antiviral effect, a pIRES empty vector control plasmid was used accordingly to maintain an equal amount of total DNA. The transfected cells were incubated for 16 h before the medium was replaced by fresh supplemented DMEM. Two days post transfection, cell culture supernatants were harvested and cleared by centrifugation at 660 g for 3 min.

Infectious HIV-1 yield in TZM-bl cells was determined as described previously (52). To determine infectious HIV-1 yield in TZM-GFP cells, 6,000 cells were seeded per well of a 96-well plate and infected the following day with 50 µL of *vpu*-deficient HIV-1 stock collected from the transfected cells. Three days post infection, cells were imaged using the Sartorius Incucyte S3/SX1—Live Cell Analysis Instrument. Images were captured as a 2 × 2 non-overlapping image montage per well in the phase contrast and green channels using the 10× objective. Image analysis was performed using the in-built analysis tools of the Incucyte (version 20211.1.7745.21572). Infectious virus yield was determined by taking the ratio of total green area per well (µm^2^/well) to the total phase contrast area (µm^2^/well) per well.

### HIV-1 p24 ELISA

Determination of the amount of *vpu*-deleted HIV-1 virions released in the supernatant and those tethered on the cell surface and present intracellularly was performed using ELISA as described previously (52). Briefly, high-binding ELISA plates (Sarstedt, #82.1581.200) were coated with the p24-coating antibody (ExBio, #11-CM006-BULK) overnight at room temperature. The following day, the coated plates were washed, blocked with 10% FCS in 1× PBS for 2 h at 37°C and washed again. The p24 protein standard and the samples were added to the wells, and the plate was incubated overnight at room temperature. The next day, the plate was washed and incubated with a polyclonal anti-HIV p24 antiserum (home-made) for 1 h at 37°C and after washing with a secondary HRP-conjugated antibody (Dianova, #111–035-008) for 1 h at 37°C. Following a wash after the incubation with the secondary antibody, TMB substrate was added and the plate was incubated in the dark at room temperature and monitored for color development. The reaction was stopped using 0.5M H_2_SO_4_ after no noticeable color change was seen for the p24 standard with the highest concentration. Absorption was measured at 450 nm with a baseline correction at 650 nm using a microplate reader.

### Flow cytometry

Flow cytometry was used to determine the cell surface and intracellular expression levels of galliform tetherin and FLAG-tagged TM-CC(aT)s in transiently transfected HEK293T cells. Additionally, HEK293T cells transiently transfected with either untagged or FLAG-tagged human tetherin expression vectors were included as controls. One day post transfection, cells were harvested and washed twice in PBS. Each sample was split into two fractions to stain either for cell surface or intracellular expression. For cell surface staining, cells were stained using either the mouse anti-human tetherin antibody (BioLegend 348410) or the rat anti-FLAG antibody (BioLegend 637307) and their matching isotype controls (mouse isotype control, BioLegend 400122 or rat isotype control, BioLegend 402305) in FACS buffer (1% FCS in PBS) for 30 min at 4°C. After staining, the cells were washed twice with the FACS buffer, fixed with 2% PFA for 30 min at 4°C, and then washed twice in the FACS buffer. The stained cells were resuspended in the FACS buffer and stored at 4°C until data acquisition. For intracellular staining, the initially aliquoted unstained fractions of cells were fixed with 2% PFA for 30 min at 4°C. After fixing, the cells were washed twice in FACS buffer and then stained using the aforementioned antibodies in FACS buffer containing 0.1% Triton X-100 for 30 min at 4°C. The permeabilized and intracellular stained cells were washed twice with FACS buffer, and finally, the cells were resuspended in FACS buffer and stored at 4°C until data acquisition. Data acquisition was performed using a Miltenyi Biotec MACSQuant VYB flow cytometer.

### Statistical analysis

For the evaluation of statistical significance, we performed Welch’s *t* tests. This test is robust to unequal standard deviations of the data sets tested. For the TMCC(aT) and tetherin experiments in human cells, the statistical significance was calculated using ordinary one-way ANOVA with Dunnett’s multiple comparison test where the mean of each column is compared to that of the 0 ng TMCC(aT) values.
